# Supplementary material for: ProteinShader: illustrative rendering of macromolecules
Source: BMC Struct Biol. 2009 Mar 30;9:19. doi: 10.1186/1472-6807-9-19 (PMC2672931; doi:10.1186/1472-6807-9-19)
Supplement: Additional file 1 — ProteinShader program without source code. This compressed file contains the complete ProteinShader program including associated libraries, but no source code. A README.txt file gives an overview of the ProteinShader distribution, and the index.html file in the help subdirectory has directions on getting started with the program as well as a set of tutorials. [file 1472-6807-9-19-S1.zip › ProteinShader-beta-0_9_4-binary/help/api/org/proteinshader/gui/utils/package-summary.html]

org.proteinshader.gui.utils (ProteinShader API)


|  |  |  |
| --- | --- | --- |
| **Overview** \| **Package** \| Class \| **Use** \| **Tree** \| **Deprecated** \| **Index** \| **Help** | |  |
| **PREV PACKAGE**   **NEXT PACKAGE** | **FRAMES**    **NO FRAMES**     **All Classes** |  |


---

## Package org.proteinshader.gui.utils

Holds minor utility classes needed by the Renderer.

**See:**
  
          **Description**

| **Class Summary** | |
| --- | --- |
| **CenteredListCellRenderer** | Centers the horizontal alignment of text in a JList or JComboBox. |
| **FileExtensionFilter** | Checks if a filename has an extension. |
| **FpsLabel** | Knows how to print a frames per second label (such as 'fps = 120') on the canvas. |
| **ImageFileChooser** | Extends JFileChooser by requiring that an ImageFormatEnum be given to the constructor. |
| **PaddedListCellRenderer** | Adds a single blank space of padding in front of the text written in a cell of a JList or a JComboBox. |
| **SeparatorListCellRenderer** | Uses a Matte Border to add a separator (a dividing line) to the top of a menu item. |

## Package org.proteinshader.gui.utils Description

Holds minor utility classes needed by the Renderer.
